# Incidences and Risk Factors of Organ Manifestations in the Early Course of Systemic Sclerosis: A Longitudinal EUSTAR Study

**DOI:** 10.1371/journal.pone.0163894

**Published:** 2016-10-05

**Authors:** Veronika K. Jaeger, Elina G. Wirz, Yannick Allanore, Philipp Rossbach, Gabriela Riemekasten, Eric Hachulla, Oliver Distler, Paolo Airò, Patricia E. Carreira, Alexandra Balbir Gurman, Mohammed Tikly, Serena Vettori, Nemanja Damjanov, Ulf Müller-Ladner, Jörg H. W. Distler, Mangtao Li, Ulrich A. Walker, EUSTAR co-authors

**Affiliations:** 1 Department of Rheumatology, University Hospital Basel, Basel, Switzerland; 2 Department of Dermatology, University Hospital Basel, Basel, Switzerland; 3 Department of Rheumatology A, Cochin Hospital, Paris Descartes University, Paris, France; 4 Department of Rheumatology, University Clinic Schleswig-Holstein, Lübeck, Germany; 5 Service de Médecine Interne, Hôpital Huriez, Université de Lille, Lille, France; 6 Department of Rheumatology, University Hospital Zurich, Zurich, Switzerland; 7 UO Reumatologia ed Immunologia Clinica, Spedali Civili, Brescia, Italy; 8 Servicio de Reumatologia, Hospital Universitario 12 de Octubre, Madrid, Spain; 9 B. Shine Rheumatology Unit, Rambam Health Care Campus, Rappaport Faculty of Medicine, Technion—Institute of Technology, Haifa, Israel; 10 Division of Rheumatology, Chris Hani Baragwanath Academic Hospital, University of the Witwatersrand, Johannesburg, South Africa; 11 Rheumatology Department, Second University of Naples, Naples, Italy; 12 Institute of Rheumatology, University of Belgrade Medical School, Belgrade, Serbia; 13 Department of Rheumatology and Clinical Immunology, Kerckhoff Clinic, Justus-Liebig University Giessen, Bad Nauheim, Germany; 14 Department of Internal Medicine 3, University of Erlangen-Nuremberg, Erlangen, Germany; 15 Department of Rheumatology, Peking Union Medical College Hospital, Chinese Academy of Medical Sciences, Beijing, China; University of Texas Health Science Center at Houston, UNITED STATES

## Abstract

**Objective:**

Systemic sclerosis (SSc) is a rare and clinically heterogeneous autoimmune disorder characterised by fibrosis and microvascular obliteration of the skin and internal organs. Organ involvement mostly manifests after a variable period of the onset of Raynaud's phenomenon (RP). We aimed to map the incidence and predictors of pulmonary, cardiac, gastrointestinal (GI) and renal involvement in the early course of SSc.

**Methods:**

In the EUSTAR cohort, patients with early SSc were identified as those who had a visit within the first year after RP onset. Incident SSc organ manifestations and their risk factors were assessed using Kaplan-Meier methods and Cox regression analysis.

**Results:**

Of the 695 SSc patients who had a baseline visit within 1 year after RP onset, the incident non-RP manifestations (in order of frequency) were: skin sclerosis (75%) GI symptoms (71%), impaired diffusing capacity for monoxide<80% predicted (65%), DU (34%), cardiac involvement (32%), FVC<80% predicted (31%), increased PAPsys>40mmHg (14%), and renal crisis (3%). In the heart, incidence rates were highest for diastolic dysfunction, followed by conduction blocks and pericardial effusion. While the main baseline risk factor for a short timespan to develop FVC impairment was diffuse skin involvement, for PAPsys>40mmHg it was higher patient age. The main risk factors for incident cardiac manifestations were anti-topoisomerase autoantibody positivity and older age. Male sex, anti-RNA-polymerase-III positivity, and older age were risk factors associated with incident renal crisis.

**Conclusion:**

In SSc patients presenting early after RP onset, approximately half of all incident organ manifestations occur within 2 years and have a simultaneous rather than a sequential onset. These findings have implications for the design of new diagnostic and therapeutic strategies aimed to ‘widen' the still very narrow ‘window of opportunity'. They may also enable physicians to counsel and manage patients presenting early in the course of SSc more accurately.

## Introduction

Systemic sclerosis (SSc) is a rare and clinically heterogeneous autoimmune disorder. Prevalence estimates vary around 20 per 100’000 [1]. The connective tissue and small vessels are mostly affected which leads to the characteristic fibrosis and vascular obliteration of the skin and internal organs, particularly of the heart, lungs, kidneys and digestive tract [2,3]. In the vast majority of individuals, SSc starts with the onset of Raynaud’s phenomenon (RP). Skin sclerosis and internal organ involvement manifest mostly either with a variable temporal interval after RP onset or simultaneously with RP.

Numerous cross-sectional studies have already assessed the prevalence of internal organ manifestations and calculated risk factors in patients with established SSc [4–9]. These studies have demonstrated that the presence of specific autoantibodies in the patient’s serum, the patient’s sex, and age at SSc onset as well as the extent of skin involvement are associated with the prevalence and severity of internal organ involvement [4–12].

As internal organ involvement constitutes an important cause of morbidity and mortality, exact data about the incidence and temporal evolution of their manifestation after RP onset are essential for physicians, who need to counsel patients and risk stratify them early after SSc diagnosis; and for investigators, who design and perform a clinical trial aimed at altering the natural course of SSc [13–15]. However, only few studies have prospectively assessed the evolution of SSc-related organ manifestations after the onset of RP. Given the paucity of reliable data, our aim was to map the incidence of internal organ manifestations early during the course of disease. By using real-life data from the large and multinational EUSTAR cohort [8,16], we assessed the acquisition of pulmonary, cardiac, gastrointestinal or renal involvement in patients who developed SSc no later than 1 year after RP onset.

## Patients and Methods

### Study population and design

The structure of the multicentre and international, prospective, longitudinal European Scleroderma Trials and Research (EUSTAR) database has been described previously [8,16]. Ethics approval according to the Declaration of Helsinki has been obtained from all respective contributing centers’ local ethics committees and ethics committee approval for the EUSTAR study was obtained from the Ethik Kommission Beider Basel (EKBB, now Ethikkommission Nordwest- und Zentralschweiz, EKNZ). Each participating centre obtained local ethics committee approval and written informed consent was required to be signed by each patient. Demographic and disease characteristics were collected between the time of the database implementation in 2004 and February, 2014. Data were considered for analysis on the condition that patients were older than 18 years at the time of the visit, and fulfilled the 1980 American College of Rheumatology (ACR) classification for SSc [17]. This dataset is hereafter called the “*entire EUSTAR cohort*”. In a second filtering step, the study population was further restricted to patients who had a baseline visit within the first year after RP onset, in order to ensure that patients were enrolled early in their disease course. This restricted dataset is hereafter called “*the study population*”.

### Study outcomes

In the *entire EUSTAR cohort*, the time between RP onset and the onset of the first non-Raynaud's manifestation of SSc (non-RP) was evaluated. In the restricted *study population*, the time between RP onset and the onset of various organ manifestations was assessed i.e. skin involvement (defined as a modified Rodnan skin score (mRSS) ≥2 points in at least 1 body area); gastrointestinal (GI) symptoms (defined as the patient reporting either dysphagia, reflux, early satiety, vomiting, diarrhoea, bloating or constipation); a systolic pulmonary artery pressure (PAPsys, as estimated by echocardiography) >40 mmHg as a proxy for suspected pulmonary hypertension; a forced vital capacity (FVC) <80% of predicted as a proxy for a pulmonary restrictive defect; digital ulcers (DU); cardiac involvement (defined as either the presence of diastolic dysfunction, conduction blocks, a left ventricular ejection fraction (LVEF) <50%, or a pericardial effusion); and lastly, renal crisis and erectile dysfunction (defined as a score <22 points in the International Index of Erectile Function (IIEF-5) questionnaire) [18]. Table 1 summarises the EUSTAR definitions of the study outcomes assessed.

**Table 1:** Definitions of study outcomes.

Study outcome	Description
Skin involvement	A modified Rodnan skin score of 2 or more points in at least 1 body area.
Gastrointestinal symptoms	Any of dysphagia, reflux, early satiety, vomiting, diarrhoea, bloating and/or constipation as reported by the patient.
Elevated systolic pulmonary artery pressure	A systolic pulmonary artery pressure as estimated by echocardiography of more than 40 mmHg as a proxy for suspected pulmonary hypertension.
Impaired forced vital capacity	A forced vital capacity of less than 80% of predicted as a proxy for a pulmonary restrictive defect.
Digital ulcers	Current ulcers distal to or at the proximal interphalangeal joint not thought to be due to trauma.
Cardiac involvement	Any of diastolic dysfunction, conduction blocks, impaired left ventricular ejection fraction and/or pericardial effusion.
Diastolic dysfunction	As estimated by echocardiography.
Conduction blocks	Atrioventricular block, bundle branch blocks as assessed by electrocardiography.
Impaired left ventricular ejection fraction	A left ventricular ejection fraction less than 50% as estimated by echocardiography.
Pericardial effusion	A pericardial effusion of 5mm or more as estimated by echocardiography.
Renal crisis	Scleroderma renal crisis as per scleroderma expert judgement.
Erectile dysfunction	A score of less than 22 points in the International Index of Erectile Function questionnaire [18].

### Statistical analysis

Frequencies and percentages as well as medians and interquartile ranges (IQR) and means and standard deviations (SD) were reported for categorical and continuous variables, respectively. Kaplan-Meier (KM) analyses were carried out to assess the cumulative probabilities of developing disease features as a function of time after RP onset for cases with available information. The date of the visit at which organ manifestations were first observed was used as the end time, i.e. the incidence. As the first visit was required to be within the first year after RP onset, manifestations that were already present at the first visit were also regarded as incident. If the manifestation was never observed, the date of the last follow up visit was set as the censor time. KM estimates were stratified by sex, age (dichotomized at the median age at RP onset), autoantibody status, and diffuse or limited skin involvement. Patients were classified as having diffuse or limited skin involvement according to their skin involvement with the first year after RP onset. Strata were compared with log-rank tests. Furthermore, incidence rates and their 95% confidence intervals (CI) were calculated. Cox proportional hazards regression analysis was used to assess the combined effect of the potential risk factors sex, age, autoantibody status and the extent of skin involvement on disease manifestations. All data were analysed with Stata 13.1 (Stata Corporation, College Station, Texas, USA).

## Results

### Patient characteristics

**Fig 1:**
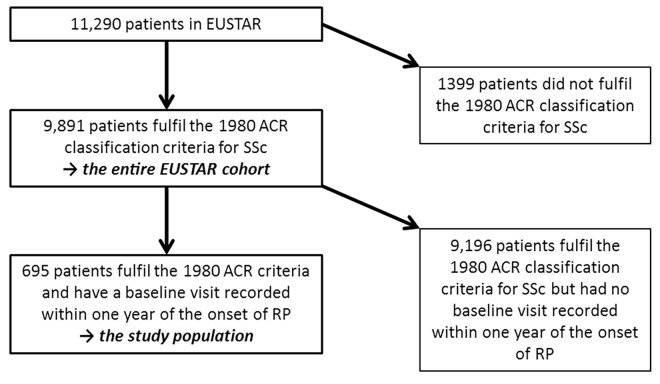
Flow chart of patients included and excluded in the analysis.

At the time of censoring, a total of 11,290 patients were followed in the EUSTAR database. Of these patients, 9,891 adult patients fulfilled the 1980 ACR classification criteria for SSc, and were therefore included in the subsequent analysis of the *entire EUSTAR cohort* [17]. The *study population*, consisting of patients with a baseline visit within the first year after RP onset, was composed of 695 subjects (Fig 1) with a median observation time of 2.1 years (IQR 0.7–4.6; mean 3.1 years, SD 3.0). The median age of *the study population* was 52.7 years (IQR 42.3–62.5) at RP onset, 27% were men (Table 2). The other patients followed in the *entire EUSTAR cohort* were on average about 9 years older and about 13% of these were male (Table 2). Compared to the *study population*, a higher percentage of the other patients followed in the *entire EUSTAR cohort* was ACA positive and a lower percentage had anti-TOPO or anti-RNAP-III autoantibodies (Table 2).

**Table 2:** Comparison of disease characteristics at the baseline visit between patients included in this analysis (visit within 1 year after onset of RP, *study population*) and those excluded (no visit within 1 year after onset of RP). ACA, anti-centromere autoantibodies; ANA, anti-nuclear autoantibodies; anti-RNAP-III, anti-RNA-polymerase-III autoantibodies; anti-TOPO, anti-topoisomerase-I autoantibodies; DLCO, single breath diffusing capacity for monoxide; FVC, forced vital capacity; IQR, interquartile range; mRSS, modified Rodnan skin score; PAPsys, systolic pulmonary artery pressure as estimated by echocardiography; RP, Raynaud's phenomenon; SD, standard deviation.

Patient characteristics at baseline visit	n^#^	Included	Excluded	P-Value
Number of patients	695	695	9196	
Age at onset of RP, years; mean (SD)	695	51.7 (14.2)	42.4 (14.8)	<0.001
Male, %	695	26.6	13.3	<0.001
**Laboratory parameters per patient**				
ANA, %	683	96.1	96.4	0.67
ACA, %	648	16.7	34.7	<0.001
Anti-TOPO, %	659	42.0	33.2	<0.001
Anti-RNAP-III*, %	317	9.5	2.9	<0.001
**Disease characteristics at baseline**				
Age at onset of first non-RP, years; mean (SD)	607	50.9 (14.4)	46.3 (14.1)	<0.001
Digital ulcers, %	684	28.4	34.2	0.002
Puffy fingers*, %	375	52.7	37.3	<0.001
mRSS; median (IQR)	643	10.0 (4.0–19.0)	6 (3.0–12.0)	<0.001
Diffuse cutaneous involvement*, %	327	20.6	9.0	<0.001
FVC*, % of predicted; mean (SD)	294	90.3 (19.3)	93.5 (21.4)	0.01
DLCO, % of predicted; mean (SD)	500	68.9 (20.8)	68.9 (20.6)	0.99
PAPsys*, mmHg; mean (SD)	242	30.2 (12.3)	30.6 (13.2)	0.63
Diastolic dysfunction, %	621	15.0	17.7	0.08
Conduction blocks, %	629	8.9	10.7	0.15
Left ventricular ejection fraction*, %; mean (SD)	284	62.8 (6.4)	62.1 (6.7)	0.12
Pericardial effusion*, %	286	8.4	6.0	0.11
Oesophageal symptoms, %	692	56.7	66.2	<0.001
Stomach symptoms, %	686	19.2	24.4	0.003
Intestinal symptoms, %	688	16.9	24.5	<0.001
Renal crisis, %	687	2.6	2.0	0.28

* Data were only captured by EUSTAR since 2007.

^#^ Number of patients with available information for each variable.

### Evolution of first non-RP manifestation of SSc in the entire EUSTAR cohort

**Fig 2:**
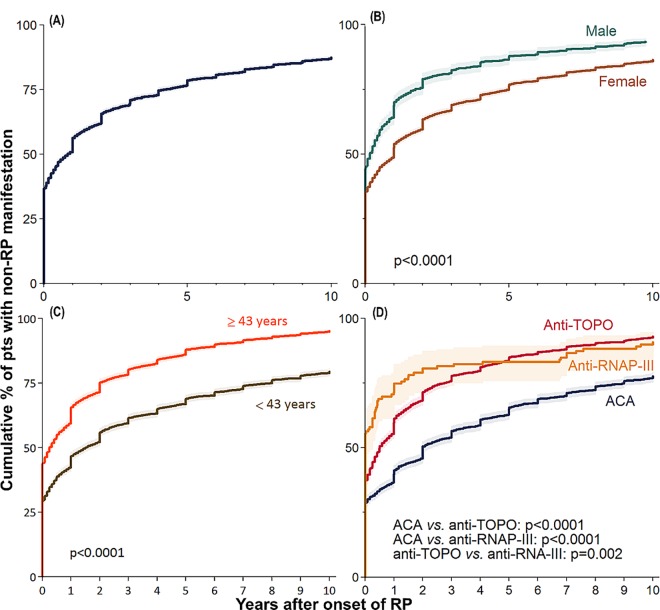
Kaplan-Meier curves with 95% CI of the manifestation of any first non-RP feature after RP onset in all SSc patients in the entire EUSTAR cohort (A) and stratified by sex (B), the median age at RP onset (C) and the autoantibody status (D). CI, confidence interval; RP, Raynaud’s phenomenon; Pts, patients; ACA, anti-centromere autoantibodies; anti-TOPO, anti-topoisomerase-I autoantibodies; anti-RNAP-III, anti-RNA-polymerase-III autoantibodies; hash marks represent censored observations.

In the *entire EUSTAR cohort* around 87% of patients had their first non-RP feature of the disease either after RP onset, or simultaneously with RP onset. The median time from RP onset until the first non-RP manifestation of SSc was 0.9 years (IQR 0–4.2), with 90% of patients acquiring their first non-RP manifestation within 12.0 years (95%CI 89.5–90.8; Fig 2A). Men developed the first non-RP manifestation faster than women (Fig 2B), and older patients were affected faster than younger patients (Fig 2C). Patients with anti-RNAP-III and anti-TOPO autoantibodies were more likely to develop a non-RP manifestation faster than patients with ACA (Fig 2D). A multivariable analysis confirmed the aforementioned risk factors for disease onset (data not shown).

### Evolution of any organ involvement in the study population

**Fig 3:**
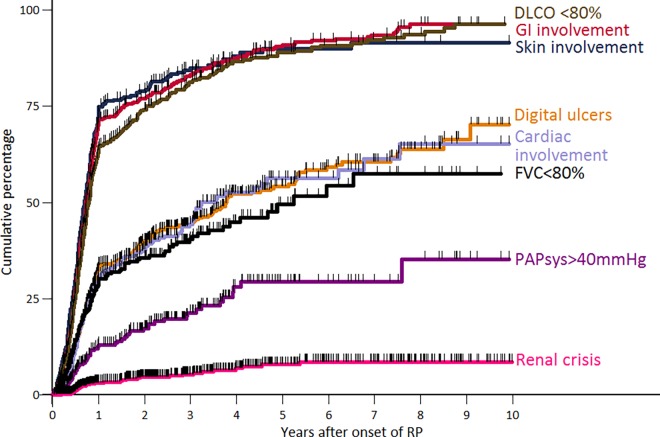
Kaplan-Meier curves of incident organ involvement in SSc patients of the study population after RP onset. RP, Raynaud’s phenomenon; Pts, patients; DLCO, single breath diffusing capacity for monoxide; GI symptoms, gastrointestinal symptoms, defined as a history of either dysphagia, reflux, early satiety, vomiting, diarrhoea, bloating or constipation; Skin involvement, defined as a modified Rodnan skin score of ≥2 at any part of the body; Cardiac involvement, defined as either the presence of diastolic dysfunction, conduction blocks, a left ventricular ejection fraction (LVEF) < 50%, or a pericardial effusion; FVC, forced vital capacity; PAPsys, systolic pulmonary artery pressure as estimated by echocardiography.

Among *the study population*, the probability of developing any organ involvement varied among the different organ systems (Fig 3). More than 90% of patients in the study population developed either a skin involvement, GI symptoms, or a single breath diffusing capacity for monoxide (DLCO)<80% of predicted, the majority of patients within the first year. The incidence rates of DU, cardiac involvement and FVC<80% of predicted were considerably lower; PAPsys>40 mmHg and renal crisis were least frequent.

#### Pulmonary complications

**Fig 4:**
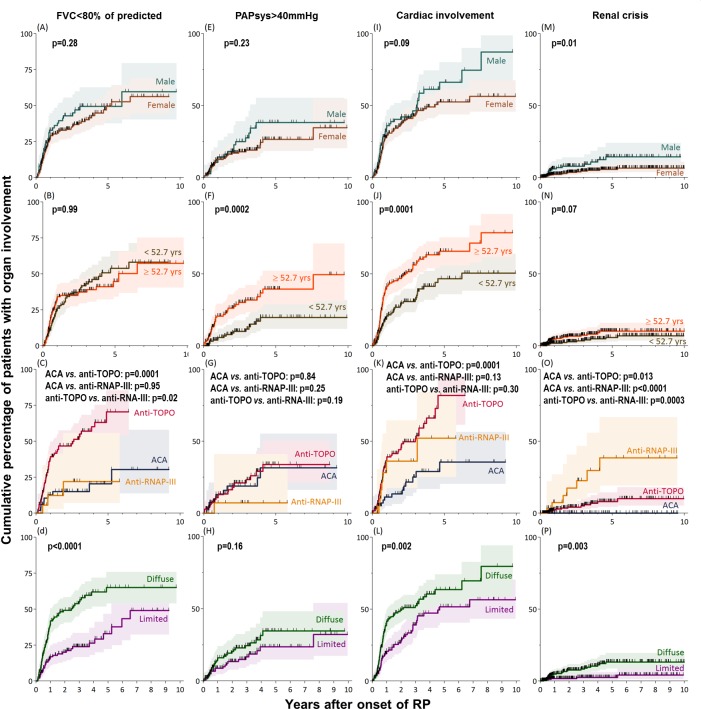
Kaplan-Meier curves with 95% CI of incident pulmonary restriction (FVC<80% of predicted; (A-D)), suspected pulmonary hypertension (PAPsys>40 mmHg; (E-H)), cardiac involvement (I-L) and renal crisis (M-P) after RP onset in SSc patients of the study population; stratified by sex (A/E/I/M), the median age at RP onset (B/F/J/N), autoantibody status (C/G/K/O) and extent of skin involvement within the first year after RP onset (D/H/L/P). CI, confidence interval; FVC, forced vital capacity; PAPsys, systolic pulmonary artery pressure as estimated by echocardiography; cardiac involvement, defined as either the presence of diastolic dysfunction, conduction blocks, a left ventricular ejection fraction (LVEF)<50%, or a pericardial effusion; RP, Raynaud’s phenomenon; yrs, years; ACA, anti-centromere autoantibodies; Anti-TOPO, Anti-topoisomerase-I autoantibodies; Anti-RNAP-III, anti-RNA-polymerase-III autoantibodies; hash marks represent censored observations.

There was no evidence that the frequency and the time to develop a FVC<80% of predicted were influenced by the patient’s sex and age (Fig 4A and 4B). There were however differences according to the serum autoantibody status (Fig 4C) as patients harbouring anti-TOPO autoantibodies had a significantly higher incidence of FVC<80% of predicted than patients with anti-RNAP-III autoantibodies (incidence ratio 3.6, 95%CI 1.2–17.9), and with ACA (incidence ratio 4.7, 95%CI 2.3–10.7). Patients with diffuse skin involvement within the first year after RP onset also more frequently had a FVC<80% of predicted than patients with limited skin involvement (incidence ratio 2.8, 95%CI 1.8–4.3; Fig 4D). In multivariable analysis, diffuse skin involvement within the first year after RP onset was the only significant risk factor for incident FVC<80% of predicted (Table 3).

**Table 3:** Cox multivariable regression analysis of risk factors for the time to incident FVC<80% of predicted, PAPsys>40 mmHg, any cardiac dysfunction, diastolic dysfunction, conduction block, pericardial effusion and renal crisis. ACA, anti-centromere autoantibodies; Anti-RNAP-III, anti-RNA-polymerase-III autoantibodies; Anti-TOPO, anti-topoisomerase-I autoantibodies; cardiac involvement, defined as either the presence of diastolic dysfunction, conduction blocks, left ventricular ejection fraction (LVEF)<50%, or pericardial effusion; CI, confidence interval; FVC, forced vital capacity; HR, hazard ratio; PAPsys, systolic pulmonary artery pressure as estimated by echocardiography; RP, Raynaud's phenomenon.

		FVC<80% of predicted		PAPsys >40mmHg		Any cardiac involvement		Diastolic dysfunction		Conduction block		Pericardial effusion		Renal Crisis	
		HR	95%CI	HR	95%CI	HR	95%CI	HR	95%CI	HR	95%CI	HR	95%CI	HR	95%CI
**Sex**															
	Male	1		1		1		1		1		1		1	
	Female	1.30	0.75–2.25	**0.40**	**0.17–0.93**	0.85	0.52–1.38	1.09	0.54–2.22	2.12	0.85–5.28	0.46	0.19–1.13	**0.39**	**0.15–0.97**
**Age at onset of RP (in years)**		1.01	0.99–1.03	**1.09**	**1.05–1.13**	**1.04**	**1.02–1.06**	**1.08**	**1.04–1.11**	1.01	0.99–1.04	1.02	0.98–1.06	**1.04**	**1.00–1.08**
**Autoantibody status**															
	ACA	1		1		1		1		1		-*	-	-*	-
	Anti-TOPO	2.37	0.80–7.03	2.04	0.61–6.80	**3.90**	**1.82–8.35**	1.78	0.61–5.20	17.59	3.81–81.33	1		1	
	Anti-RNAP-III	0.62	0.13–2.91	0.49	0.05–4.76	2.56	0.91–7.19	2.09	0.55–8.00	6.62	0.88–49.95	0.31	0.04–2.31	**5.18**	**2.03–13.22**
**Extent of skin involvement**															
	Limited	1		1		1		1		1		1		1	
	Diffuse	**3.08**	**1.43–6.62**	0.91	0.33–2.50	0.93	0.52–1.64	1.78	0.72–4.38	**0.45**	**0.21–0.95**	2.86	0.65–12.51	2.96	0.68–12.81

* no patient with ACA developed a renal crisis or pericardial effusion.

A FVC<50% of predicted (i.e. a severe pulmonary restrictive defect) was diagnosed in 2% (95%CI 1–5) of patients within the first year and in 12% (95%CI 6–23) during the 10-year follow up.

The probability to develop a DLCO<80% of predicted was high (Fig 3). In multivariable analysis, anti-TOPO positivity as well as a diffuse skin involvement were the main risk factors for incident DLCO<80% of predicted (HR 1.60, 95%CI 1.12–2.28; HR 1.52, 95%CI 1.14–2.02, respectively).

In the first 3 years after RP onset, about one third of patients acquired a DLCO<50% of predicted (95%CI 27–36), with a progressive increase to 54% of patients (95%CI 44–65) during the observational period. Older patients, male patients, patients with anti-TOPO autoantibodies, or with diffuse skin involvement had a higher incidence of DLCO<50% of predicted (incidence ratios: older *vs*. younger 1.8, 95%CI 1.3–2.5; male *vs*. female 1.9, 95%CI 1.4–2.6; anti-TOPO *vs*. ACA 3.6, 95%CI 2.0–6.49 diffuse *vs*. limited 2.9, 95%CI 2.0–4.2). However, only older age, the presence of anti-TOPO autoantibodies and diffuse skin involvement were confirmed risk factors for developing a DLCO<50% of predicted in multivariable analysis (HR 1.03 per 1 year increase of age, 95%CI 1.01–1.04; HR 2.64, 95%CI 1.23–5.64; HR 2.06, 95%CI 1.19–3.56, respectively).

The time to develop a PAPsys>40 mmHg was not associated with the patient’s sex or the patient’s extent of skin involvement (Fig 4E and 4H). Older patients however acquired a PAPsys>40 mmHg faster and more frequently than younger patients (Fig 4F). Patients with anti-RNAP-III autoantibodies showed a PAPsys>40 mmHg less frequently than patients harbouring ACA or anti-TOPO autoantibodies, but there was no statistically significant difference (Fig 4G). Patient’s older age was confirmed to be the main risk factor for developing PAPsys>40 mmHg (Table 3).

#### Cardiac involvement

There was no evidence that a patient’s sex was associated with the time to cardiac involvement (Fig 4L). Older patients, however, had a 2.1-fold higher incidence (95%CI 1.5–3.0; Fig 4J) and patients with diffuse skin involvement had a 1.9-fold higher incidence (95%CI 1.3–2.7) of cardiac involvement than patients with limited skin involvement (Fig 4L). Patients with anti-TOPO autoantibodies developed cardiac involvement more frequently and more rapidly than patients with ACA (incidence ratio 3.7, 95%CI 2.0–7.4; Fig 4K). In multivariable analysis older age and the presence of anti-TOPO autoantibodies remained risk factors for any cardiac involvement (HR 1.04 per 1 year increase of age and 3.90, respectively; Table 3).

**Fig 5:**
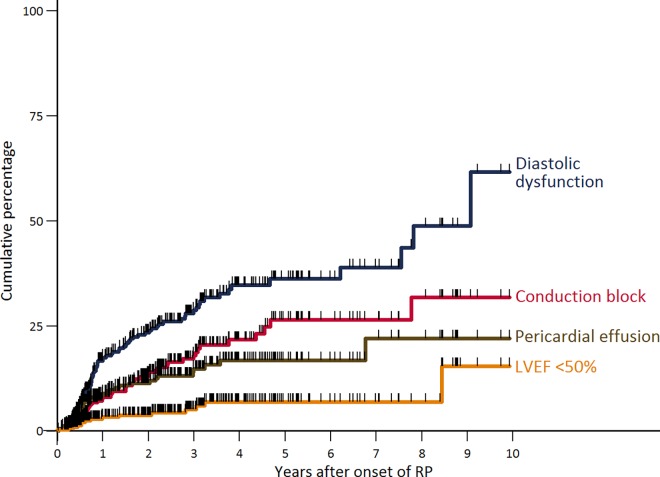
Kaplan-Meier curves of incident cardiac manifestations after RP onset in SSc in the study population. RP, Raynaud’s phenomenon; Pts, patients; LVEF, left ventricular ejection fraction; hash marks represent censored observations.

The most common manifestation of cardiac involvement was diastolic dysfunction (Fig 5). The incidence of diastolic dysfunction did not differ between sexes and autoantibody status (incidence ratios: male *vs*. female 1.0, 95%CI 0.6–1.6; ACA *vs*. anti-TOPO 0.5, 95%CI 0.2–1.1; ACA *vs*. anti-RNAP-III 0.5, 95%CI 0.2–1.8); though the incidence of diastolic dysfunction was 3.5 times (95%CI 2.1–5.9) higher in older than in younger patients. The frequency and the time to develop a diastolic dysfunction were also influenced by the extent of skin involvement with diffuse patients having a 2.1-fold higher incidence (95%CI 1.3–3.4). In the multivariable analysis, however, only older age remained a risk factor for diastolic dysfunction (HR 1.08 per 1 year increase of age; Table 3).

Conduction blocks were the second most frequent type of heart involvement (Fig 5). The incidence did neither vary by patient’s sex, patient’s age, nor the patient's extent of skin involvement; but patients with anti-TOPO autoantibodies had a considerably higher incidence of conduction blocks than patients with ACA (incidence ratio 10.0, 95%CI 2.5–86.2). In multivariable analysis, patients with anti-TOPO autoantibodies and patients with RNAP-III autoantibodies had an increased risk for conduction blocks, although for the latter, this was not statistically significant (Table 3).

Pericardial effusion was one of the less frequent types of heart involvement. This complication evolved more frequently and more rapidly in men, compared to women (incidence ratio 2.7, 95%CI 1.4–5.4) and in patients with diffuse compared to limited skin involvement (incidence ratio 5.6, 95%CI 2.3–16.5). There was no evidence for a difference between age strata. It is noteworthy, that none of the patients with ACA developed a pericardial effusion, compared to 26% (95%CI 17–39) of patients with anti-TOPO autoantibodies. In multivariable analysis, neither patient’s sex nor the extent of skin involvement were confirmed as significant risk factors (Table 3).

During the first 3 years after RP onset, a LVEF<50% was only observed in 5% of patients (95%CI 3–9; Fig 5).

#### Gastrointestinal symptoms

GI symptoms was one of the most common disease features; at baseline 57% of patients reported oesophageal symptoms, 19% stomach symptoms and 17% intestinal symptoms. There was no evidence for a difference in the cumulative percentages of GI symptoms when stratifying by sex, or autoantibody status (p = 0.66 and p = 0.16, respectively). However, older patients and patients with diffuse skin involvement tended to acquire a GI symptoms earlier and more frequently (p = 0.02 and p = 0.02, respectively). In multivariable analysis, older age was a borderline risk factor (HR 1.01 per 1 year increase in age, 95%CI 1.00–1.02) in contrast to diffuse skin involvement (diffuse *vs*. limited: HR 1.35, 95%CI 1.04–1.76); in addition, patients with anti-RNAP-III were less likely to develop GI symptoms than patients with ACA or anti-TOPO autoantibodies (HR 0.55, 95%CI 0.34–0.90; HR 0.59, 95%CI 0.39–0.91, respectively).

#### Urogenital involvement

Around 3% (95%CI 2–5) of patients developed a renal crisis within 1 year after the onset of RP (Fig 3). The majority of patients who developed a renal crisis did so within the first 4 years. Men had a 2.5 times (95%CI 1.2–5.2) higher incidence than women (Fig 4M). There was no difference between younger and older patients (Fig 4N). The cumulative percentage of renal crisis varied markedly between the autoantibody groups (Fig 4O). Patients with anti-RNAP-III autoantibodies had a 4.6 times (95%CI 1.6–12.4) higher incidence of renal crisis than patients harbouring anti-TOPO and none of the patients with ACA developed a renal crisis. Patients with diffuse skin involvement developed a renal crisis earlier and more frequently than patients with a limited skin involvement (Fig 4P). In multivariable analysis male sex, anti-RNAP-III positivity, and older age conferred independent risk factors for renal crisis (Table 3).

The IIEF-5 questionnaire was not included in the EUSTAR database at its inauguration. As a consequence, data on erectile dysfunction was only available for 17% (n = 32) of men in the study population. Around 52% (95%CI 36–70) of these patients reported an erectile dysfunction (IIEF-5 score<22) during the first 3 years after RP onset progressing to 95% (95%CI 79–100) during the subsequent 6 years. In the first 3 years after onset of RP, about 30% (95%CI 18–52) of the men had developed a severe erectile dysfunction (IIEF-5 score<8). Owing to the small number of men with data on erectile dysfunction, no further stratification was feasible.

## Discussion

This longitudinal study uniquely analysed the incidence of organ manifestations in a large cohort of SSc patients early after RP onset and details differences in the evolution of organ involvement. Whereas other investigators have analysed the risk factors for disease manifestation in patients at risk for SSc (“pre-SSc”, “very early SSc”), the present study has focused on the analysis of incident organ manifestations in established disease [19,20].

Skin sclerosis, symptoms of GI tract and a reduced pulmonary diffusing capacity were frequent complications of early SSc, incident cardiac complications and pulmonary restriction were observed more rarely, followed by elevated systolic pressures of the pulmonary artery and renal crisis. Our study also highlights a high incidence of diastolic dysfunction, whereas other cardiac complications (conduction blocks, pericardial effusion and left ventricular systolic dysfunction) were less frequent [21].

In every organ system analysed, approximately half of all organ manifestations that occurred during the 10 year observation period became evident within the first 2 years after RP onset. Thus, the disease onset followed a simultaneous rather than sequential manifestation pattern. Regardless of the differences in the observed frequencies, i.e. the height of the cumulative incidences, of these manifestations, the steep increase in manifestations during the first two years after RP onset were persistently observed across all organs manifestations studied; even complications which are regarded as more severe were not restricted to later disease. Another important point is that approximatively 75% of the patients develop organ involvement during the 5 first years of the disease. This is good news for the patients who reach that point without any organ involvement.

In line with retrospective prevalence estimates, there were differences in the risk factors governing the onset of organ complications [8,11,22–26]. These risk factors modified the cumulative incidences of the organ manifestations but did not substantially modify the steep increase in manifestation rates during the first 2 years after RP onset. This observation, together with the short interval between RP onset and the first non-RP manifestation demonstrates rapid initial disease kinetics and suggests a relatively short ‘window of opportunity’ to prevent incident organ damage [27]. Other investigations have also suggested that a variety of severe organ complications (pulmonary hypertension and lung fibrosis, among others) are not restricted to late disease [25,28].

The large number of SSc patients and the longitudinal and multinational setup of this study are strengths of this investigation. Our study also uniquely simulated an inception cohort by including only patients into the *study population* who had a baseline visit within the first year after RP onset. At the same time we introduced a selection bias, as evidenced by the high prevalence of factors commonly associated with more prevalent and severe organ complications (male sex, older age at SSc onset and anti-TOPO autoantibodies) [8,10,22,29,30]. This patient selection could account for the previously unreported association of male sex and renal crisis identified in this study and underlines that our findings are specific to SSc patients who present with SSc early after RP onset and must not be generalized to all individuals who present with SSc. However, as also demonstrated in this study, more than half of all patients in the *entire EUSTAR cohort* experienced their first non-RP feature of the disease within one year of the onset of RP. It must be also noted, that some SSc patients had documented organ manifestations already at their baseline visit, leading to an overestimation of the time to its onset. Lastly, our data may be biased by centre specific differences in the assessment of some organ manifestations. With regard to diastolic dysfunction for example, different diagnostic approaches are available, each differing in sensitivity and specificity and predictive value [31]. Also, the in the EUSTAR database the DLCO collected is not corrected for haemoglobin.

Despite these limitations, our data will likely improve the counselling and management of SSc patients early after RP onset. Our findings also have implications for the design of new diagnostic strategies and therapeutics aimed to ‘widen’ the still very narrow ‘window of opportunity’ [27].

## Supporting Information

S1 File EUSTAR co-authors. (PDF)
