# Supplementary material for: Incidences and Risk Factors of Organ Manifestations in the Early Course of Systemic Sclerosis: A Longitudinal EUSTAR Study
Source: PLoS One. 2016 Oct 5;11(10):e0163894. doi: 10.1371/journal.pone.0163894 (PMC5051961; doi:10.1371/journal.pone.0163894)
Supplement: S1 File — (PDF) [file pone.0163894.s001.pdf]

EUSTAR co-authors - Lidia Ananieva, Stefan Heitmann, Simona Rednic, Valeria Riccieri, Magdalena Szmyrka-Kaczmarek, Dominique Farge, Giovanni Lapadula, Marco Matucci-Cerinic, Serena Guiducci, Nicolas Hunzelmann, Massimo Ricci, Carina Mihai, Douglas Veale, Roger Hesselstrand, Eduardo Mariok, Vanessa Smith, Ingo H. Tarner, Eugene J. Kucharz, László Czirják, Duska Martinovic, Kamal Solanki, Codrina Mihaela Ancuta, Jean Sibilia, Caramaschi Paola, Manal Hassanien, Sarah Kahl, Frederick Wigley, Marie Vanthuyne, Daniela Opris, Sebastião C. Radominski, Andrea Lo Monaco, Ada Corrado, Michaela Koehm, Veronica Codullo, Bevcar Radim, Esthela Loyo, Maria Üprus, Raffaele Pellerito, Thierry Zenone, Armando Gabrielli, Otylia Kowal-Bielecka, Blaz Rozman, Raffaella Scorza, Lesley Ann Saketkoo, Oyvind Midtvedt, Carlos Alberto von Mühlen, Jörg Henes, Ani Branimir, Paul Hasler, Sule Yavuz, Sabine Adler, Brigitte Krummel-Lorenz, Magdalena Posa, Merete Engelhart, Christopher Denton, Dorota Krasowska, Paloma Garcia de la Peña Lefebvre, Franco Cozzi, Luc Mouthon, Edoardo Rosato, Carlo Selmi, Juan José Alegre Sancho, Carmel Mallia, Massimiliano Limonta, Matthias Seidel, Rosario Foti, Lisa Stamp, Susanne Ullman, Simon Stebbings, Vera Ortiz Santamaria, Francesco Del Galdo, Ellen De Langhe, Alessandro Mathieu, Cord Sunderkötter, Kilian Eyerich, Bojana Stamenkovic, Srdan Novak, Percival D. Sampaio-Barros, Cristiane Kayser, Ira Litinsky, Maura Couto
